# Supplementary figures and images for: N-chlorination mediates protective and immunomodulatory effects of oxidized human plasma proteins
Source: eLife. 2019 Jul 12;8:e47395. doi: 10.7554/eLife.47395 (PMC6650281; doi:10.7554/eLife.47395)

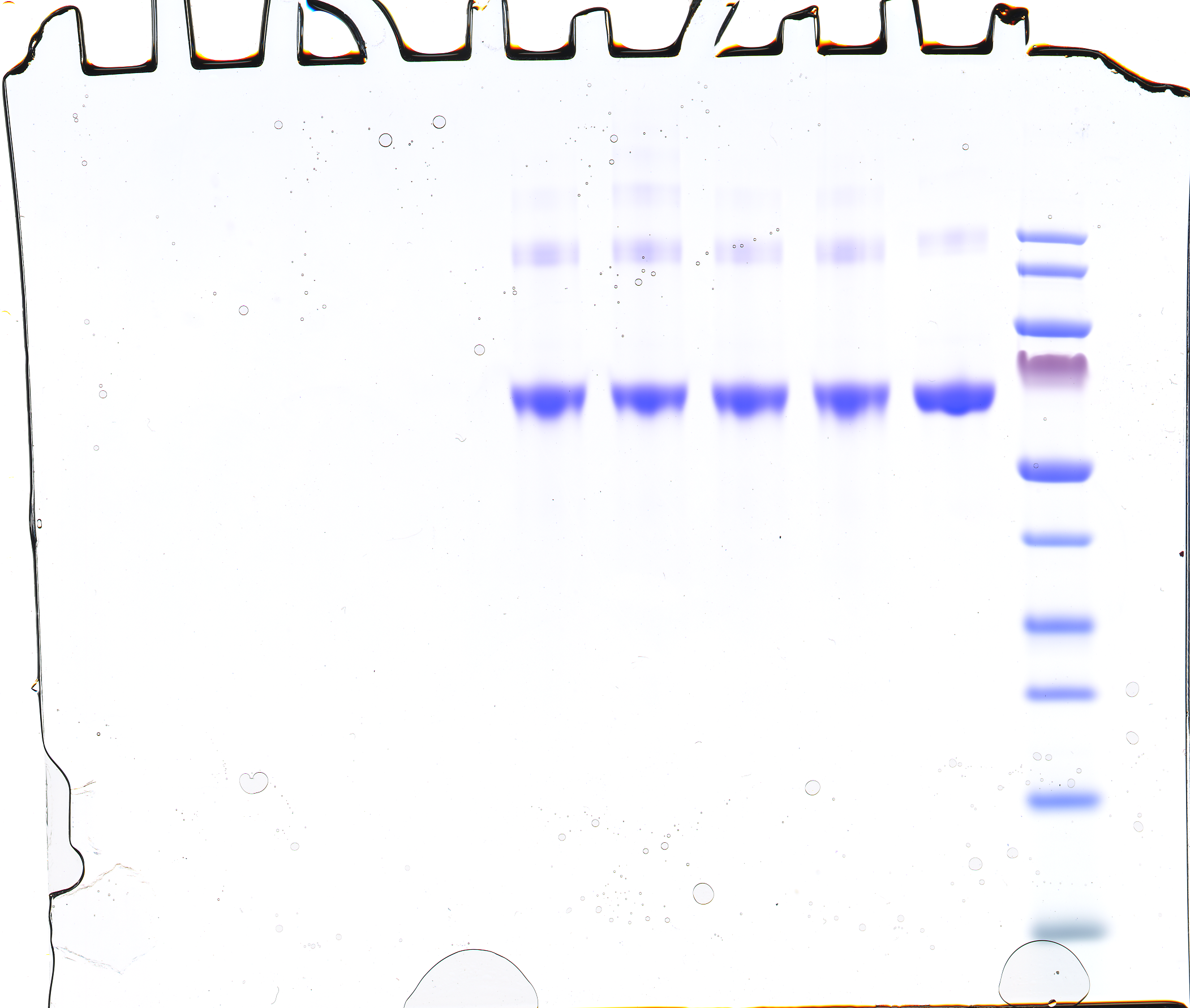

Supplement: Figure 2—figure supplement 3—source data 1. [file elife-47395-fig2-figsupp3-data1.zip › Figure 2 Source Data 8/Figure 2 figure supplement 3 b.tif]

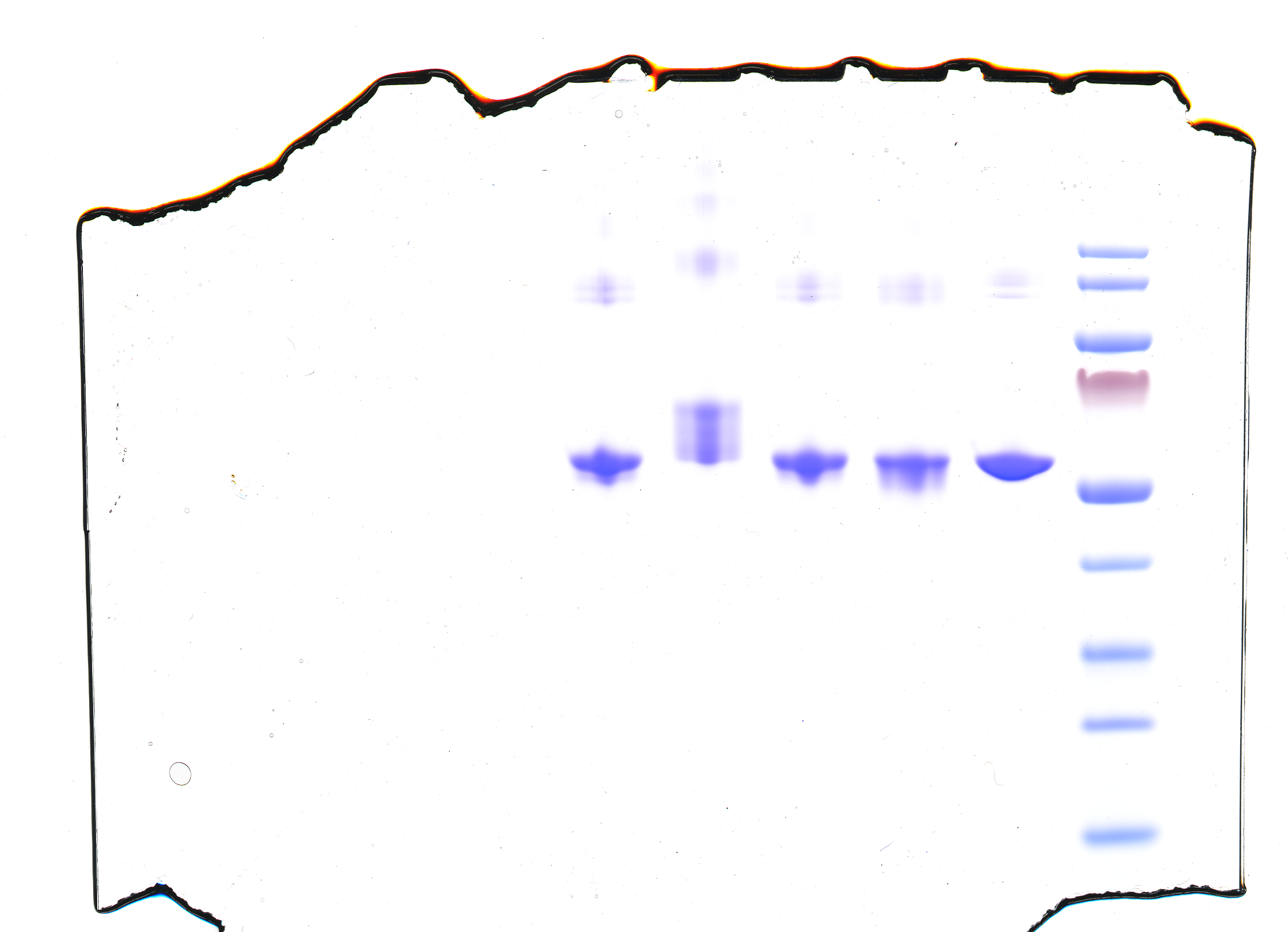

Supplement: Figure 2—figure supplement 3—source data 1. [file elife-47395-fig2-figsupp3-data1.zip › Figure 2 Source Data 8/Figure 2 figure supplement 3 a.tif]
